# High Cleavage Efficiency of a 2A Peptide Derived from Porcine Teschovirus-1 in Human Cell Lines, Zebrafish and Mice

**DOI:** 10.1371/journal.pone.0018556

**Published:** 2011-04-29

**Authors:** Jin Hee Kim, Sang-Rok Lee, Li-Hua Li, Hye-Jeong Park, Jeong-Hoh Park, Kwang Youl Lee, Myeong-Kyu Kim, Boo Ahn Shin, Seok-Yong Choi

**Affiliations:** 1 Department of Biomedical Sciences, Chonnam National University Medical School, Gwangju, Republic of Korea; 2 Research Institute of Medical Sciences, Chonnam National University Medical School, Gwangju, Republic of Korea; 3 Department of Biology, Chosun University, Gwangju, Republic of Korea; 4 Research Institute of Kim and Jung Co. Ltd., Hwasun, Republic of Korea; 5 College of Pharmacy and Research Institute of Drug Development, Chonnam National University, Gwangju, Republic of Korea; 6 Department of Pathogen Biology, Hainan Medical University, Haikou, People's Republic of China; 7 Department of Neurology, Chonnam National University Medical School, Gwangju, Republic of Korea; Kantonal Hospital St. Gallen, Switzerland

## Abstract

When expression of more than one gene is required in cells, bicistronic or multicistronic expression vectors have been used. Among various strategies employed to construct bicistronic or multicistronic vectors, an internal ribosomal entry site (IRES) has been widely used. Due to the large size and difference in expression levels between genes before and after IRES, however, a new strategy was required to replace IRES. A self-cleaving 2A peptide could be a good candidate to replace IRES because of its small size and high cleavage efficiency between genes upstream and downstream of the 2A peptide. Despite the advantages of the 2A peptides, its use is not widespread because (i) there are no publicly available cloning vectors harboring a 2A peptide gene and (ii) comprehensive comparison of cleavage efficiency among various 2A peptides reported to date has not been performed in different contexts. Here, we generated four expression plasmids each harboring different 2A peptides derived from the foot-and-mouth disease virus, equine rhinitis A virus, *Thosea asigna* virus and porcine teschovirus-1, respectively, and evaluated their cleavage efficiency in three commonly used human cell lines, zebrafish embryos and adult mice. Western blotting and confocal microscopic analyses revealed that among the four 2As, the one derived from porcine teschovirus-1 (P2A) has the highest cleavage efficiency in all the contexts examined. We anticipate that the 2A-harboring cloning vectors we generated and the highest efficiency of the P2A peptide we demonstrated would help biomedical researchers easily adopt the 2A technology when bicistronic or multicistronic expression is required.

## Introduction

In biomedical research, the simultaneous expression of more than one gene in cells or organisms using a single plasmid is sometimes required. To this end, several strategies have been employed: (i) multiple promoters fused to the genes' open reading frames (ORFs); (ii) insertion of splicing signals between genes; fusion of genes whose expressions are driven by a single promoter; (iii) insertion of proteolytic cleavage sites between genes; and (iv) insertion of internal ribosomal entry sites (IRESs) between genes [1], [2].

Of these strategies, IRES has been widely used due to the following advantages: (i) ensured coexpression of genes before and after the IRES; (ii) feasibility of adding subcellular localization sequences to the gene after IRES; and (iii) availability of commercial expression plasmids harboring IRES [2]. However, IRES has two major limitations [2], [3]. First, the size of IRES is usually longer than 500 nucleotides, which could be a problem when a large insert is cloned into IRES-containing viral vectors with limited cloning capacity or when multiple IRESs are used to generate multicistronic plasmids. Second, translation efficiency of a gene placed after the IRES is much lower than that of a gene located before IRES.

**Figure 1:**
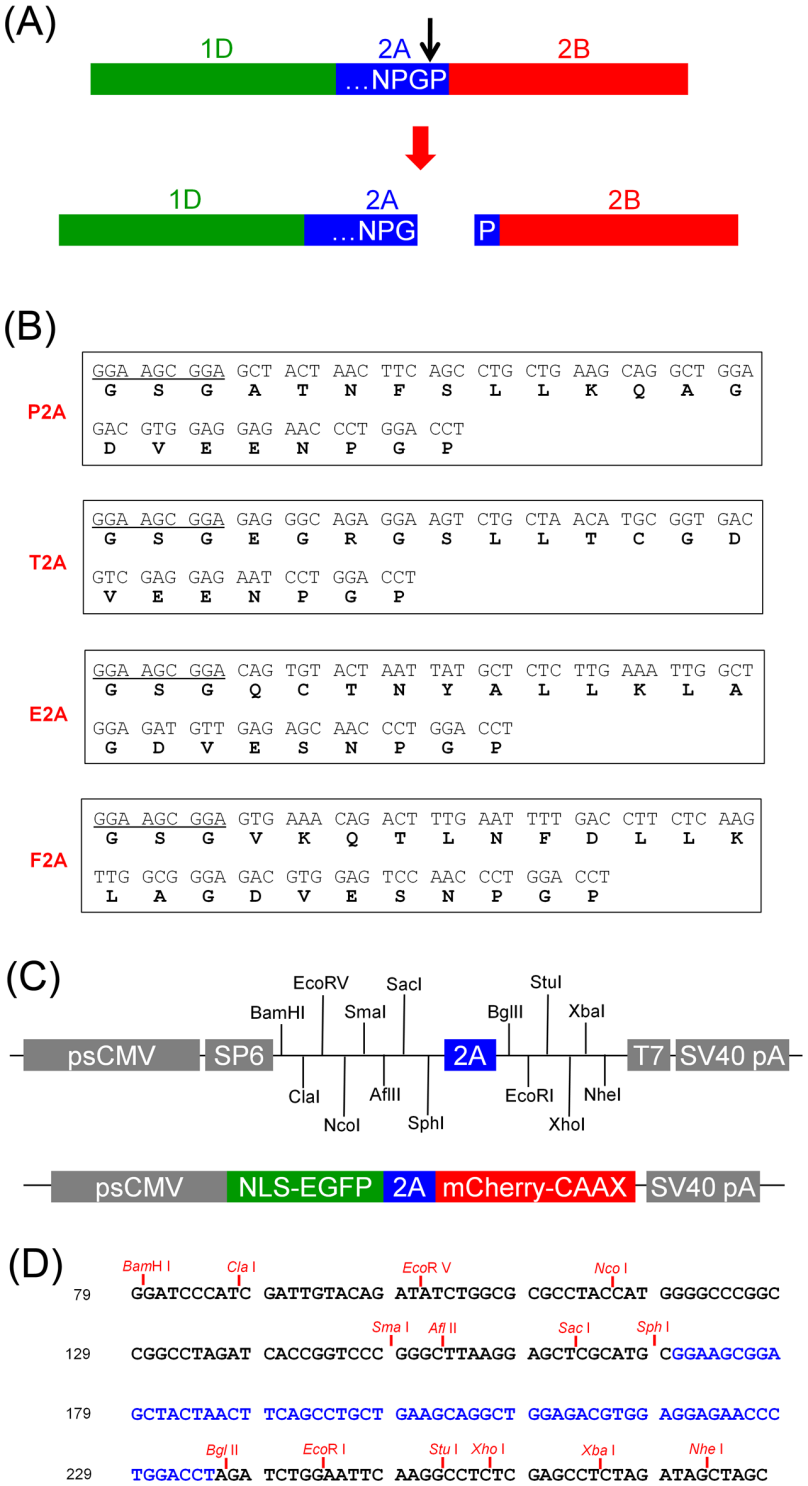
Construction of expression plasmids harboring DNA sequences encoding various 2A peptides flanked by multiple cloning sites. (A) Schematic representation of cleavage occurring in a peptide translated in foot-and-mouth disease virus (FMDV). 1D, 2A and 2B indicate contiguous endogenous peptides translated in FMDV. The arrowhead indicates cleavage site. (B) DNA and corresponding amino acid sequences of various 2A peptides. Underlined sequences encode amino acids GSG, which were added to improve cleavage efficiency. P2A indicates porcine teschovirus-1 2A; T2A, *Thoseaasigna* virus 2A; E2A, equine rhinitis A virus (ERAV) 2A; F2A, FMDV 2A. (C) A schema showing a map for an expression plasmid harboring DNA sequences encoding a 2A peptide (upper) and a construct used in this study encoding 2A peptides flanked by NLS-EGFP and mCherry-CAAX. NLS and CAAX denote the nuclear localization sequence and membrane localization sequence, respectively. (D) Sequences of the multiple cloning sites and recognition sites of unique restriction endonucleases therein. Nucleotides in blue encode a P2A peptide.

These limitations can be overcome by a 2A peptide, a “self-cleaving” small peptide first identified by Ryan and colleagues in the foot-and-mouth disease virus (FMDV), a member of the picornavirus [4]. The average length of 2A peptides is 18–22 amino acids. The designation “2A” refers to a specific region of picornavirus polyproteins and arose from a systematic nomenclature adopted by researchers. In FMDV, it is delineated at its own C-terminus by ‘cleavage’ (to be more precise, by ribosome skipping) and at its N-terminus by a proteolytic cleavage or ‘trimming’ from the upstream capsid protein 1D by the 3C/3CD proteinase. Initially, it was speculated that either a virus-encoded proteinase or host cell proteinase might be responsible for the cleavage [4]. Recent reports demonstrated, however, that ribosomes skip the synthesis of the glycyl-prolyl peptide bond at the C-terminus of a 2A peptide, leading to the cleavage between a 2A peptide and its immediate downstream peptide [5], [6], [7]. As a result, the cleaved-off downstream peptide has proline at its N-terminus (Fig. 1A). The term CHYSEL (*cis*-acting hydrolase element) was coined by Pablode Felipe to reflect the characteristics of this type of picornavirus 2A peptides [8].

2A-mediated cleavage is a universal phenomenon in all eukaryotic cells. However, it has not been observed in prokaryotic cells [9]. In addition to the FMDV 2A peptide, several 2A peptides have since been identified in picornaviruses, insect viruses and type C rotaviruses [2]. Of the 2A peptides identified to date, four have been widely used in biomedical research: FMDV 2A (abbreviated herein as F2A); equine rhinitis A virus (ERAV) 2A (E2A); porcine teschovirus-1 2A (P2A) and *Thoseaasigna* virus 2A (T2A). The former three viruses belong to picornaviruses and the latter is an insect virus [3].

Two advantages of the 2A peptide over IRES are its short length and stoichiometric expression of multiple proteins flanking the 2A peptide [3]. Despite these advantages, the use of 2A peptides has not been as common in biomedical research as it should be; we speculated that there might be two reasons. First, it is not certain which 2A peptide has the highest cleavage efficiency. Donnelly and colleagues showed in 2001 that T2A has the highest cleavage efficiency (close to 100%) followed by E2A, P2A and F2A [10]. In addition, Szymczak and colleagues demonstrated in 2004 that F2A and T2A have higher efficiency (close to 100%) than E2A [11]. However, since their claims were based on *in vitro* transcription/translation experiments, it remains unclear as to how the 2A peptides would act in an *in vivo* context. Second, to the best of our knowledge, there is no publicly or commercially available cloning vector harboring a ubiquitous promoter and a 2A gene flanked by multiple cloning sites, which could make it difficult for scientists to adopt 2A technology for coexpression of more than one gene.

To circumvent these two obstacles, we first set out to compare the cleavage efficiency of F2A, E2A, P2A and T2A peptides in three human cell lines, zebrafish and mice using Western blotting and confocal microscopy analyses. Second, we embarked on constructing cloning vectors bearing a simian cytomegalovirus (sCMV) promoter fused to a 2A gene flanked by multiple cloning sites.

## Materials and Methods

### Ethics statement

Animal studies were approved by the Chonnam National University Medical School Institutional Animal Care and Use Committee (Project number: CNUIACUC-H-2010-20).

### Reagents

All chemicals were purchased from Sigma (MO, USA), unless indicated otherwise.

### Plasmid construction

Oligonucleotides encoding P2A, T2A, E2A or F2A (refer to Fig. 1B for sequences) were purchased from Bioneer (Daejeon, Korea), annealed and then individually cloned into SphI/BglII sites of a pCS4+ plasmid (provided by Chang-Yeol Yeo). Oligonucleotides used are as follows. P2A: 5′-CGGAAGCGGAGCTACTAACTTCAGC CTGCTGAAGCAGGCTGGAGACGTGGAGGAGAACCCTGGACCTA-3′ (forward) and 5′-GATCT AGGTCCAGGGTTCTCCTCCACGTCTCCAGCCTGCTTCAGCAGGCTGAAGTTAGTAGCTCCGCTTCCGCATG-3′ (reverse). T2A: 5′-CGGAAGC GGAGAGGGCAGAGGAAGTCTGCTAACATGCGGTGACGTCGAGGAGAAT CCTGGACCTA-3′ (forward) and 5′-GATCTAGGTCCAGGATTCTCCTCGACGTC ACCGCATGTTAGCAGACTTCCTCTGCCCTCTCCGCTTCCGCATG-3′ (reverse). E2A: 5′-CGGAAGCGGACAGTGTACTAATTATGCTCTCTTGAAATTGGCT GGAGATGTTGAGAGCAACCCTGGACCTA-3′ (forward) and 5′-GATCTAGGTCC AGGGTTGCTCTCAACATCTCCAGCCAATTTCAAGAGAGCATAATTAGTACA CTGTCCGCTTCCGCATG-3′ (reverse). F2A: 5′-CGGAAGCGGAGTGAAACAG ACTTTGAATTTTGACCTTCTCAAGTTGGCGGGAGACGTGGAGTCCAAC CCTGGACCTA-3′ (forward) and 5′-GATCTAGGTCCAGGGTTGGACTCCACGTCTCCCGCCAACTTGAGAAGGTCAAAATTCAAAGTCTGTTTCACTCCGCTTCC GCATG-3′ (reverse). The resulting construct was termed pCS4+-2A. NLS-EGFP and mCherry-CAAX were PCR-amplified and then ligated into the ClaI/AflII and BglII/NheI sites of pCS4+-2A, respectively, to produce pNLS-EGFP-2A-mCherry-CAAX. NLS and EGFP indicate the nuclear localization sequence and enhanced green fluorescent protein, respectively. All plasmids constructed were verified by digestion with restriction endonucleases (NEB; MA, USA) and DNA sequencing (Macrogen; Daejeon, Korea).

### Cell culture and transfection

HeLa, HT1080 and HEK293T cells were purchased from American Type Culture Collection (VA, USA). HeLa and HEK293T cells were cultured in Dulbecco's Modification of Eagle's Medium (DMEM; Welgene, Korea) supplemented with 10% fetal bovine serum (FBS; GIBCO, USA). HT1080 cells were cultured in DMEM (Welgene) supplemented with 10% FBS (GIBCO). Transfection of the cells with plasmids was carried out using FuGENE HD (Roche, Switzerland) according to the manufacturer's instructions.

### Western blotting (WB)

M-PER mammalian protein extraction reagent (Thermo Scientific, USA) was used to lyse the human cell lines 24 hr post-transfection, zebrafish embryos at 24 hour post-fertilization (hpf) and mouse liver 3 day post-injection (dpi). Each lysate was separated by 12% sodium dodecyl sulfate polyacrylamide gel electrophoresis (SDS-PAGE) and transferred to nitrocellulose membrane (Pall Corporation; NY, USA). Subsequently, the membrane was probed with the indicated primary antibody (anti-EGFP [1∶1000, Santa Cruz Biotechnology, catalog # sc-9996] and anti-DsRed [1∶1000, Clontech, catalog # 632393]), washed with TBST (0.2 M Tris, 1.37 M NaCl,0.1% Tween-20, pH7.6), probed with HRP-conjugated goat anti-mouse antibody (1∶4000, Santa Cruz Biotechnology, catalog # sc-2005). The bound antibody was detected by enhanced chemiluminescence (AniGen, Korea) and then exposed to X-ray film (AGFA, Belgium).

Because anti-DsRed antibody has been successfully used to decorate mCherry protein [12], anti-DsRed antibody was used to visualize the mCherry protein.

### Synthesis of RNA for microinjection into zebrafish embryos

Plasmids encoding NLS-EGFP-2A-mCherry-CAAX were linearized with ClaI and *in vitro* transcribed with SP6 RNA polymerase using an mMessage mMachine kit (Ambion). The resulting RNA (200 pg) was microinjected into one cell stage embryos.

### Generation of recombinant adenovirus

Recombinant adenovirus was generated as described previously [13]. In brief, a DNA fragment encoding NLS-EGFP-2A-mCherry-CAAX was cloned into the NotI/SalI sites of a pShuttle vector (Agilent Technologies, CA, USA). The resulting construct was linearized with PmeI and then introduced via electroporation into BJ5183 electroporation-competent cells harboring pAdEasy-1 (Agilent Technologies). The resulting recombinant clones were selected by kanamycin and digestion with restriction endonucleases. Subsequently, the verified clone was amplified, linearized with PacI and then transfected into HEK293 A packaging cells. The recombinant adenovirus was released from the cells through four freeze-thaw-vortex cycles 14–20 day post-transfection, amplified through infection of HEK293 A packaging cells and purified by CsCl ultracentrifugation. The resulting high-titer (tissue-culture infected dose at 50% [TCID_50_]) recombinant adenovirus was injected into mice via the tail vein.

### Confocal microscopy

Human cell lines were fixed 24 hr post-transfection in 4% paraformaldehyde, permeabilized in 0.2% Triton X-100 and mounted with Vectashield mounting media containing 4′,6-diamidino-2-phenylindole (DAPI; Vector Labs; CA, USA). Zebrafish embryos at 24 hpf were anesthesized intricane (Sigma) and mounted with 3% methylcellulose. Mouse liver was extracted 3 day post-injection, embedded in Tissue-Tek O.C.T. compound (Sakura Finetechnical; Tokyo, Japan), frozen rapidly in liquid nitrogen, sectioned into 10 µl in thick slices using a LeicaCM3050 microtome (Walldorf, Germany) and mounted on coverslips with Vectashield mounting media containing DAPI. The mounted samples were imaged using anLSM 510 confocal microscope (Zeiss;Hamburg, Germany), with a 100× or a 40× objective lens. The excitation wavelengths applied were 488 nm for EGFP and 543 nm for mCherry.

### Image analysis

The images taken were assembled using Adobe Photoshop and band intensity on the Western blots was analyzed using ImageJ.

## Results

To generate versatile cloning vectors harboring genes encoding viral 2A peptides, we inserted four different 2A sequences (P2A, T2A, E2A and F2A; Fig. 1B) [11] into a pCS4+ plasmid, which has a simian cytomegalovirus (sCMV) promoter, SP6 and T7 RNA polymerase binding sites, multiple cloning sites (MCSs) and an SV40 polyadenylation site [14]. The sCMV promoter is a ubiquitous promoter and can function in *Xenopus*, zebrafish, mouse and human [15]. There sulting vectors have 2A sequences flanked by several unique recognition sites for restriction endonucleases (Fig. 1C). Nucleotide sequences encoding Gly-Ser-Gly were added to the 5′end of the 2A sequences to improve cleavage efficiency [11]. Sequences of the MCSs and recognition sites of unique restriction endonucleases therein are shown in Fig. 1D.

**Figure 2:**
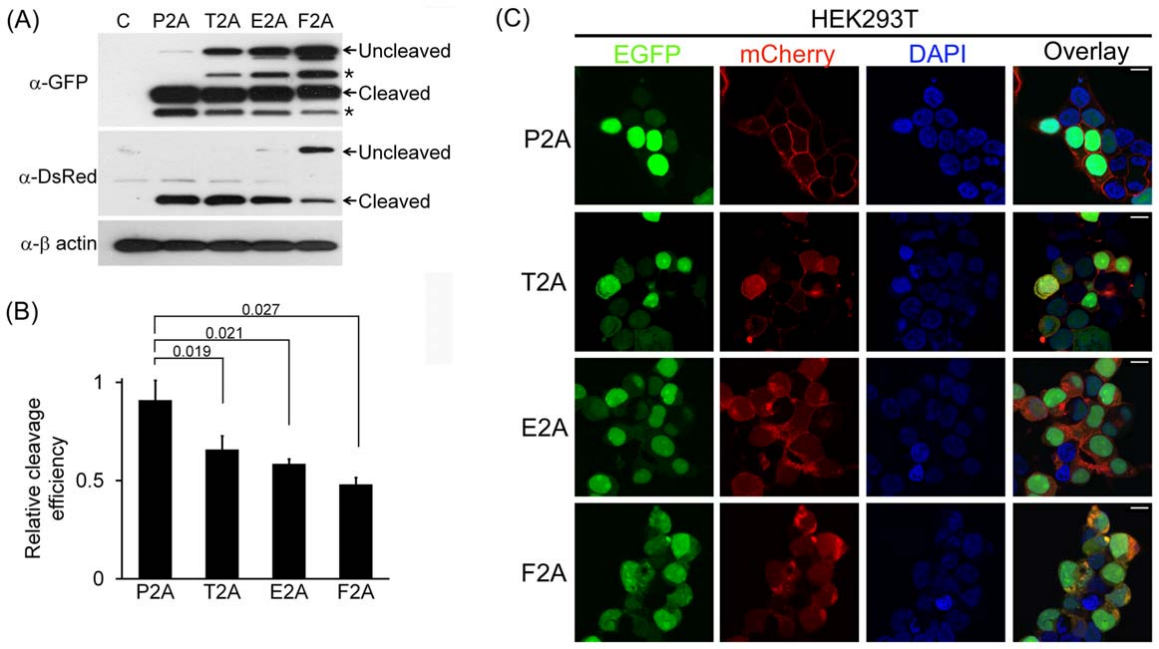
P2A shows the highest cleavage efficiency in HEK293T cells. HEK293T cells were transfected with the indicated plasmids. (A) WB analysis of cleavage efficiency of the 2As in HEK293T cells. The transfected cells were processed for WB 24 hr post-transfection. The cleavage efficiency was assessed using GFP and DsRed antibodies to decorate NLS-EGFP and mCherry-CAAX, respectively. Asterisks indicate two major byproducts. Anti-β actin antibody was used as a loading control. (B) Quantitation of cleavage efficiency of indicated 2As. Cleavage efficiency = cleaved form/(cleaved form+uncleaved form). The amount of each form was estimated from its band intensity on the Western blot measured by ImageJ software. The *p* value was determined by the two-tailed Student's *t*-test (n = 3). (C) Confocal microscopy of the transfected cells. Green signals in the nucleus that do not overlap with red signals indicate cleaved NLS-EGFP. Conversely, red signals in the plasma membrane that do not overlap with green signals represent cleaved mCherry-CAAX. In the overlay images, yellow signals derived from overlapping of green and red signals denote uncleaved NLS-EGFP-2A-mCherry-CAAX. The scale bar represents 20 µm.

**Figure 3:**
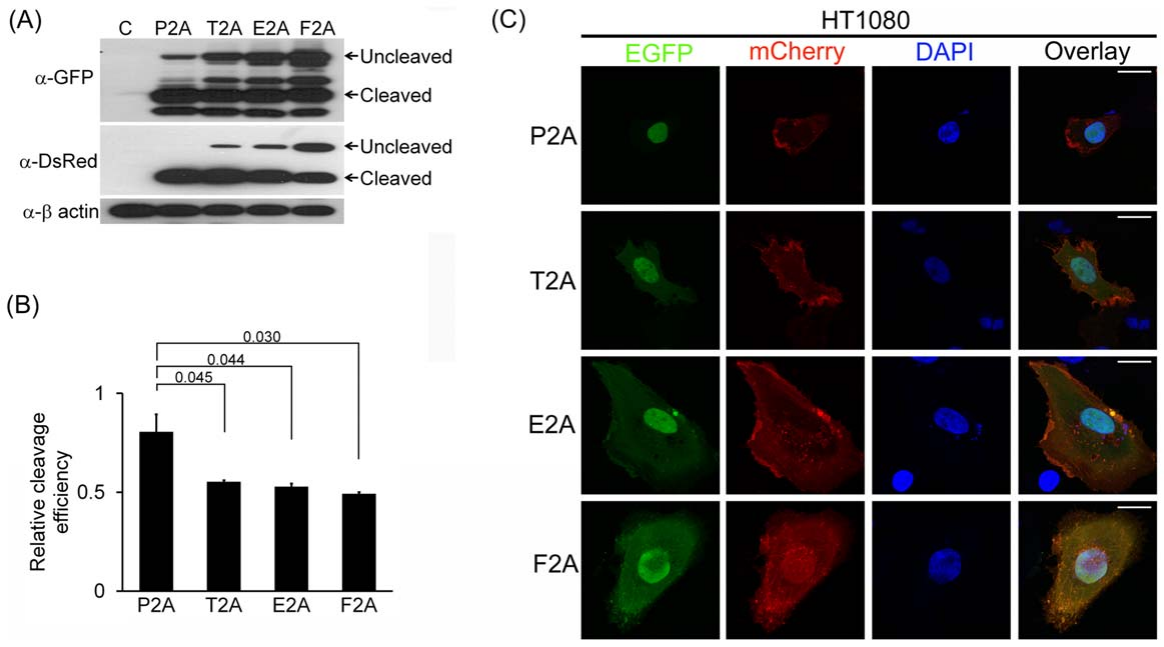
P2A shows the highest cleavage efficiency in HT1080 cells. (A–C) HT1080 cells were transfected with indicated plasmids and analyzed as described in Figure 2. The scale bar represents 20 µm.

**Figure 4:**
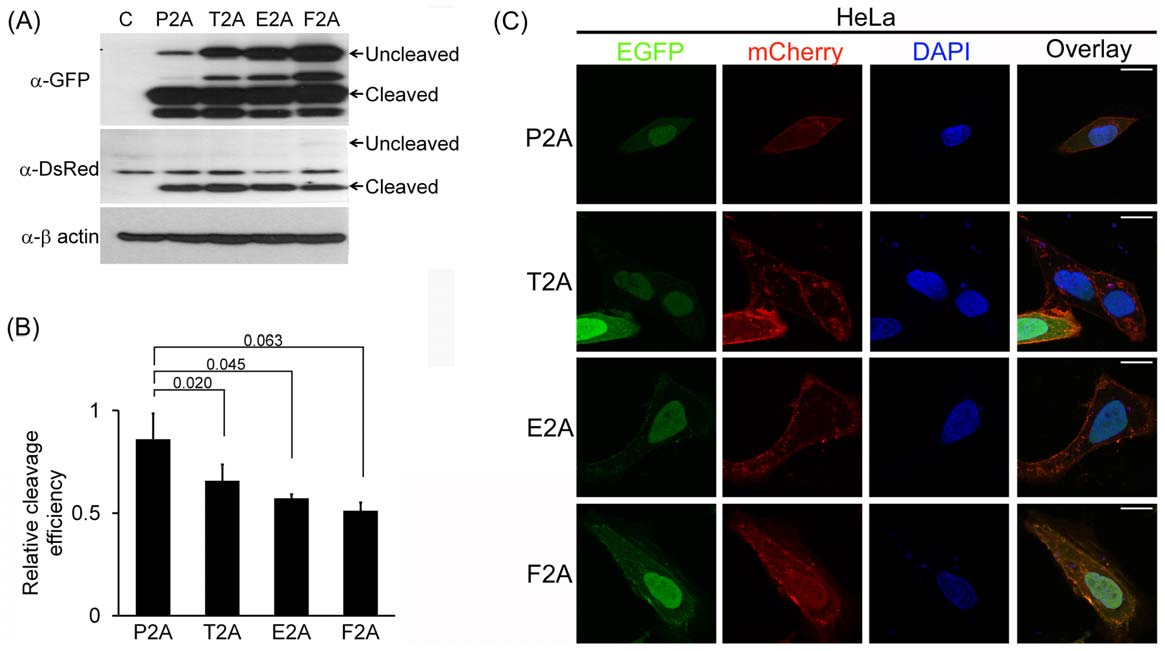
P2A shows the highest cleavage efficiency in HeLa cells. (A–C) HeLa cells were transfected with indicated plasmids and analyzed as described in Figure 2. The scale bar represents 20 µm.

To test whether the four 2A sequences used act properly, we fused a nuclear localization sequence (NLS) to the N-terminus of EGFP (termed NLS-EGFP) and a membrane localization sequence (CAAX, where X = any amino acid) to the C-terminus of mCherry (termed mCherry-CAAX) and then inserted the resulting NLS-EGFP and mCherry-CAAX [16] into the four different 2A cloning vectors generated. The resulting constructs were named pNLS-EGFP-2A-mCherry-CAAX. Subsequently, we transfected three widely used human cells lines (HEK293T [an embryonic kidney cell line], HT1080 [a fibrosarcoma cell line] and HeLa [a cervical cancer cell line]) with the pNLS-EGFP-2A-mCherry-CAAX and analyzed the cleavage efficiency of the respective 2A peptides using WB and confocal microscopy 24-hr post-transfection. WB analysis revealed that all four 2As function with various cleavage efficiency in the HEK293T (Figs. 2A and 2B), HT1080 (Figs. 3A and 3B) and HeLa cell lines (Figs. 4A and 4B), with P2A to be the most efficient, followed by T2A, E2A and F2A. The two major byproducts indicated by asterisks in Figure 2A were noted as well in a previous report [17]. Of note, the byproducts did not appear in the lysate of cells transfected with pEGFP-N1 plasmid that does not have 2A sequences (marked GFP in Fig. S1), raising the possibility that their production may be related to 2A mediated ‘cleavage’ activity.

In addition, the difference in cleavage efficiency among the 2As was confirmed by confocal microscopy: targeting of NLS-EGFP and mCherry-CAAX produced by 2A-mediated cleavage to the nucleus and plasma membrane, respectively, in the cell lines was the most efficient in P2A, followed by T2A, E2A and F2A. (Figs. 2C, 3C and 4C for HEK293T, HT1080 and HeLa cells, respectively).

**Figure 5:**
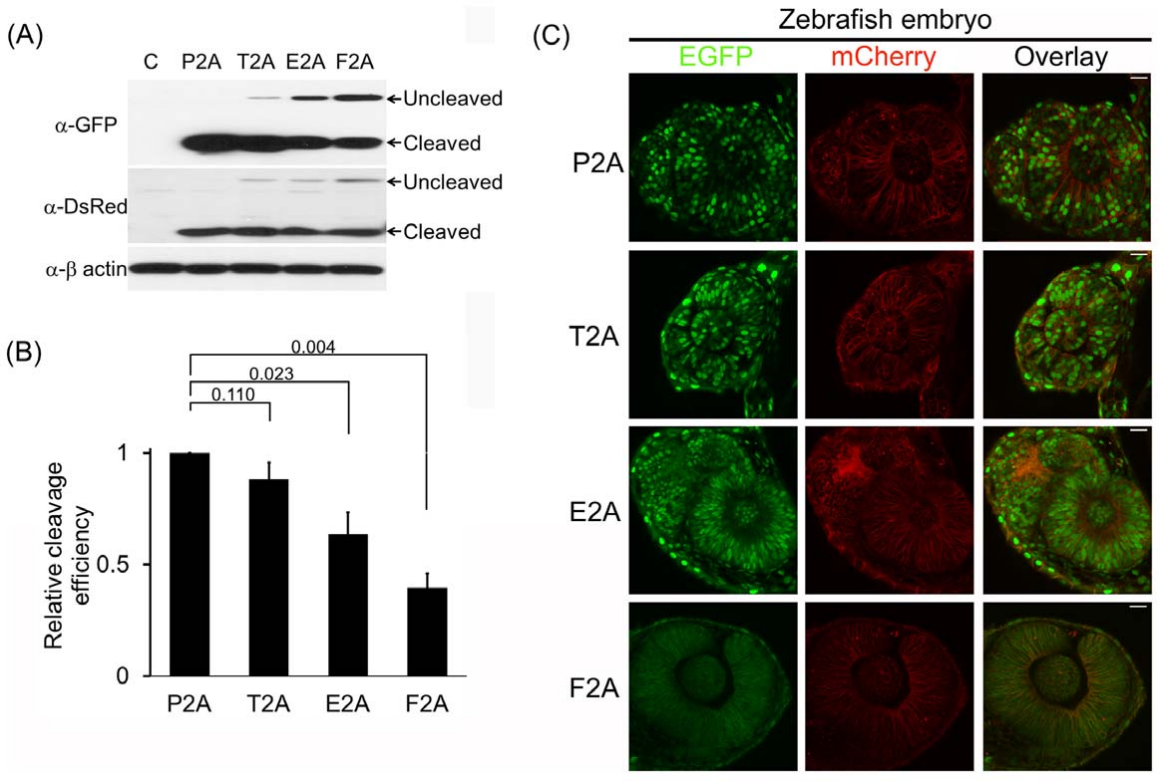
P2A shows the highest cleavage efficiency in zebrafish embryos. One- or two-cell stage embryos were injected with *in vitro* transcribed RNAs encoding the indicated proteins. (A) WB analysis revealing cleavage efficiency in the 2As in zebrafish embryos. The injected embryos at 24 hpf were processed for WB. Anti-β actin antibody was used as a loading control. (B) Quantitation of cleavage efficiency of the indicated 2As. The cleavage efficiency was calculated as depicted in Figure 2B. *P* value was determined by the two-tailed Student's *t*-test (n = 3). (C) Confocal microscopy of the retina in the injected embryos at 24 hpf. Non-overlapping green signals in the nucleus and red signals in the plasma membrane correspond to cleaved NLS-EGFP and mCherry-CAAS, respectively. Yellow signals in the overlay images signify uncleaved NLS-EGFP-2A-mCherry-CAAX. The scale bar represents 20 µm.

To determine if the results gained from experiments with the human cultured cell lines are recapitulated in zebrafish embryos, we generated mRNAs from pNLS-EGFP-2A-mCherry-CAAX using SP6 RNA polymerase and microinjected the resulting mRNAs into one-cell stage embryos. The embryos at 24 hpf were then harvested and analyzed using WB and confocal microscopy. WB analysis exhibited that P2A has the highest cleavage efficiency in zebrafish as well, followed by T2A, E2A and F2A (Fig. 5A). Moreover, confocal microscopic analysis of zebrafish embryonic retina confirmed the WB result, as judged by the targeting of NLS-EGFP and mCherry produced by 2A-mediated cleavage to their respective destination, just as shown for human cultured cell lines (Fig. 5B). Taken together, findings of the experiments with zebrafish embryos recapitulated those of human cultured cell line experiments.

**Figure 6:**
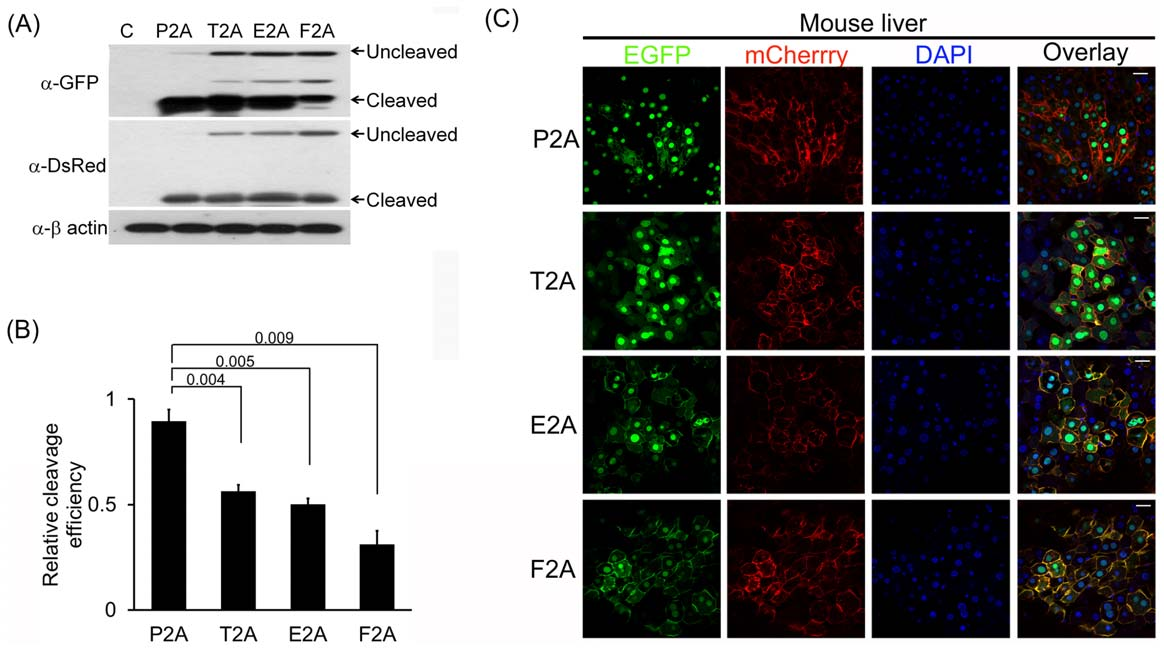
P2A shows the highest cleavage efficiency in the mouse liver. Adult mice were injected with recombinant adenovirus encoding indicated proteins via the tail vein. (A) WB analysis revealing cleavage efficiency of the 2As in the mouse liver. The liver was extracted from the injected mice at 3 day post-injection (dpi) and processed for WB. Anti-β actin antibody was used as a loading control. (B) Quantitation of cleavage efficiency of the indicated 2As. The cleavage efficiency was calculated as depicted in Figure 2b. The *p* value was determined by the two-tailed Student's *t*-test (n = 3). (C) Confocal microscopy of liver section of the injected mice. The images were taken at 3 dpi. Green, red and yellow signals are as described in the legend for Figure 5C. The scale bar represents 20 µm.

To investigate if the outcome of human cultured cell line and zebrafish embryo experiments is echoed in mouse tissue, we subcloned NLS-EGFP-2A-mCherry-CAAX into the adenoviral genome via homologous recombination, transduced packaging cell lines with the resulting recombinant adenovirus and amplified infectious adenovirus harboring pNLS-EGFP-2A-mCherry-CAAX. Subsequently, the infectious virus particles were injected into the tail veins of mice and livers were harvested 3 dpi. The harvested livers were then analyzed by WB and confocal microscopy. WB analysis displayed that P2A also has the highest cleavage efficiency in the mouse liver, followed by T2A, E2A and F2A (Fig. 6A). Furthermore, NLS-EGFP and mCherry produced by P2A-mediated cleavage were targeted most efficiently in the mouse liver as well, as evidenced by confocal microscopy (Fig. 6B). Collectively, our findings from the human cultured cell line and zebrafish embryo experiments were reiterated in the mouse liver: P2A has the highest cleavage efficiency, as demonstrated by WB and confocal microscopy.

## Discussion

Here, we show that among four different 2A peptides, a 2A peptide derived from porcine teschovirus-1 (P2A) has the highest cleavage efficiency in three human cell lines, zebrafish embryos and mouse liver. In addition, we generated sCMV promoter driven expression cloning vectors and adenoviral shuttle vectors, both harboring a 2A gene flanked by multiple cloning sites. These vectors can be used to simultaneously express multiple genes in mammalian cell lines, zebrafish and mice. Although we did not test if this is the case, it is very likely that the cloning vectors would act in a similar manner in *Xenopus*, because an sCMV promoter functions in *Xenopus* as well [15] and the 2A-mediated cleavage occurs in all eukaryotic cells [9].

What is the mechanism by which P2A peptide exhibits the highest cleavage efficiency in human cell lines, zebrafish and mouse? Ryan and colleagues proposed that there are two ways to raise the cleavage efficiency [3]. First, insertion of a GSG linker at the N-terminus of a 2A peptide can improve cleavage efficiency [11], [18]. Second, addition of C-terminal amino acids of a 1D peptide to the N-terminus of a 2A peptide can increase cleavage efficiency, as shown by the following two reports. Ryan and colleagues reported that introduction of C-terminal five amino acids (APVKQ) of an FMDV 1D peptide to the N-terminus of an FMDV 2A peptide increased the efficiency by 13% *in vitro* [9]. Groot Bramel-Verheije and colleagues demonstrated that the introduction of C-terminal seven amino acids (APVKQLL) of an FMDV 1D peptide to the N-terminus of an FMDV 2A peptide raised the efficiency by 15% in both baby hamster kidney (BHK-21) cells and porcine alveolar macrophages [19]. These two reports, however, do not account for the difference in the cleavage efficiency among 2A peptides derived from different viruses in that the reports examined the cleavage efficiency of the same 2A peptides in the presence or absence of extra amino acids originating from the 1A peptide. We have demonstrated that the 19-amino acid P2A elicits higher cleavage efficiency than the 22-amino acid 2As. This suggests that when comparing the cleavage efficiency among 2A peptides derived from different viruses, the length of 2A peptide is not the only factor that determines the cleavage efficiency. Further work is required to determine the factor(s) governing the difference in cleavage efficiency among different 2As.

We believe that identification of a highly efficient 2A peptide and construction of expression plasmids with a 2A gene flanked by multiple cloning sites would facilitate the use of 2A technology for simultaneous expression of multiple genes.

## Supporting Information

Figure S1 **Two major byproducts decorated by anti-GFP antibody in lysate of cells transfected with 2A plasmids do not appear in lysate of cells transfected with pEGFP-N1.** HEK293T cells were individually transfected with the indicated 2A plasmids and pEGFP-N1 that does not harbor 2A sequences. The transfected cells were processed for WB 24 hr post-transfection. The cleavage efficiency was assessed using anti-GFP antibodies to decorate NLS-EGFP. Asterisks indicate the two major byproducts. Note that the byproducts do not appear in lysates of cells transfected with pEGFP-N1 (GFP). Anti-β actin antibody was used as a loading control.
(TIF)
