# Supplementary figures and images for: High Cleavage Efficiency of a 2A Peptide Derived from Porcine Teschovirus-1 in Human Cell Lines, Zebrafish and Mice
Source: PLoS One. 2011 Apr 29;6(4):e18556. doi: 10.1371/journal.pone.0018556 (PMC3084703; doi:10.1371/journal.pone.0018556)

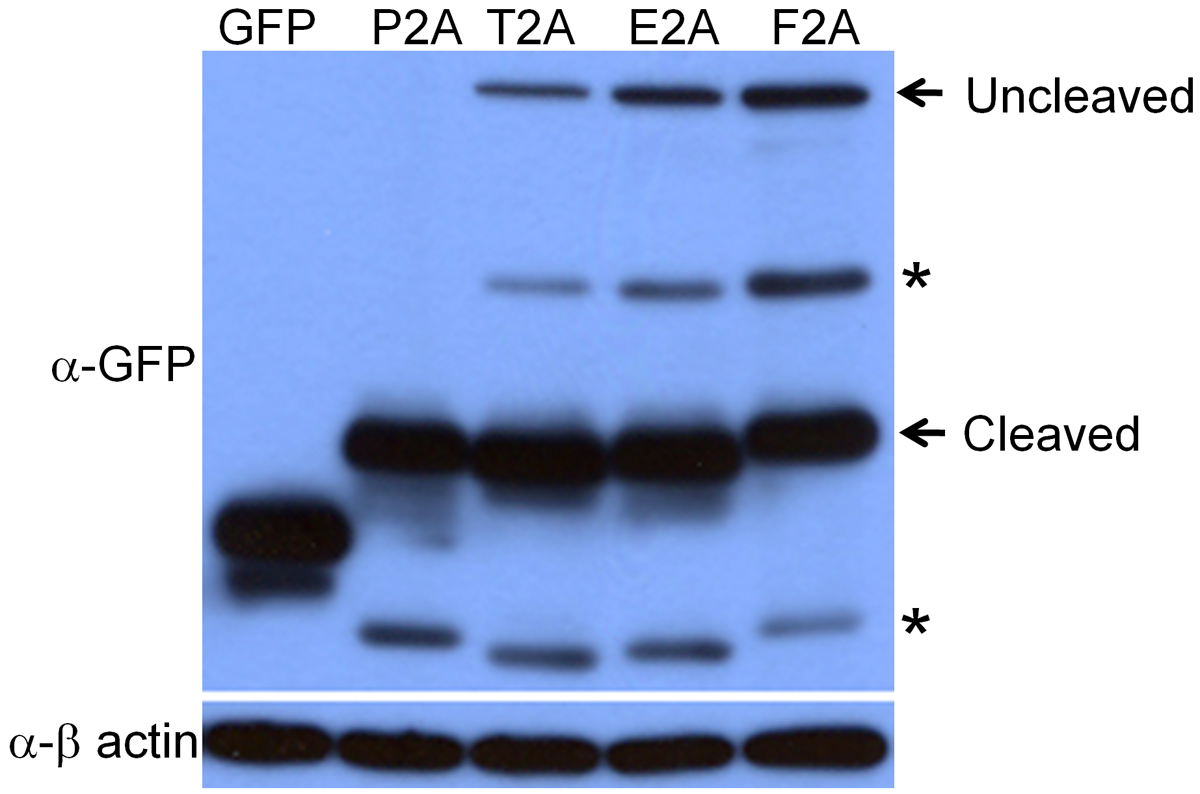

Supplement: Figure S1 — Two major byproducts decorated by anti-GFP antibody in lysate of cells transfected with 2A plasmids do not appear in lysate of cells transfected with pEGFP-N1. HEK293T cells were individually transfected with the indicated 2A plasmids and pEGFP-N1 that does not harbor 2A sequences. The transfected cells were processed for WB 24 hr post-transfection. The cleavage efficiency was assessed using anti-GFP antibodies to decorate NLS-EGFP. Asterisks indicate the two major byproducts. Note that the byproducts do not appear in lysates of cells transfected with pEGFP-N1 (GFP). Anti-β actin antibody was used as a loading control. (TIF) [file pone.0018556.s001.tif]
